# Volleyball Movement Standardization Recognition Model Based on Convolutional Neural Network

**DOI:** 10.1155/2023/6116144

**Published:** 2023-01-25

**Authors:** Baiyu Li, Ming Tian

**Affiliations:** Department of Physical Education and Research of Lanzhou University, Lanzhou, Gansu, China

## Abstract

Artificial intelligence and deep learning have attracted much attention from researchers in industry and academia. The volleyball movement standardization and recognition model involve the application of artificial intelligence and deep learning. In order to solve the problem that human action in volleyball video is continuous and effective spatial and temporal features need to be extracted from the video stream, the Inception module is decoupled and heterogeneous, replacing the original 5 × 5 convolutional structures with two 3 × 3 convolutional structures, as well as replacing the 3 × 3 convolutional structures with 1 × 3 and a 3 × 1 convolutional structure with internal parameter optimization to ensure the accuracy of recognition. The model uses the input motion video RGB map as the spatial input and the optical flow map as the temporal input, and the two are weighted 1 : 1 for feature fusion. Experiments are conducted on the volleyball action video and homemade dataset in UCF101, and the experimental data show that the accuracy of the DNet volleyball action standardization recognition model proposed in this paper is 94.12%, which proves that the method improves the recognition ability of the model while speeding up the training speed. The research presented in this paper provides important theoretical guidance for artificial intelligence and deep learning.

## 1. Introduction

Artificial intelligence and deep learning have attracted much attention from researchers in industry and academia. The volleyball movement standardization and recognition model involve the application of artificial intelligence and deep learning. The research presented in this paper provides important theoretical guidance for artificial intelligence and deep learning. Based on the computer vision platform to achieve volleyball action standard recognition, which is an important trend in the application and development of artificial intelligence in sports with a very wide range of application prospects, the surveillance camera in real-time under the human action recognition technology support to identify the volleyball player's action standardization domain and record and make a warning can effectively improve the volleyball player's sports efficiency and volleyball sports level. In addition, through the analysis of the volleyball sports video, it can be clearly derived from the video whether player action standardization, volleyball sports tactical layout, and point-to-point analysis have an important role.

With the increasing maturity of convolutional neural network technology, human action recognition based on convolutional neural networks is widely concerned by domestic and foreign research scholars. Technical action detection in sports videos is an important application in the field of computer vision in sports, among which volleyball has distinct technical characteristics and has significant advantages for individual human action technology recognition and classification. Through the recognition and classification of both players' action techniques in sports videos, it can effectively improve the technical analysis ability and technical level of volleyball players. Therefore, the convolutional neural network volleyball action standard type recognition model has important research significance and application value.

**Figure 1:**
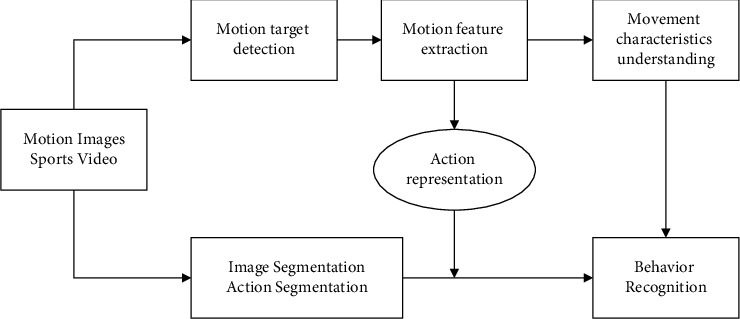
Flow chart of standard types of motion action recognition for artificial intelligence and deep learning.

For artificial intelligence and deep learning, in convolutional neural network-based volleyball action criterion recognition models, the extraction of effective action features as well as representation features from video sequences is an important part of action analysis, retrieval, and recognition, which directly affects the accuracy and robustness of its results. In the past decades, many video action recognition methods and action recognition datasets have emerged. With the successful application of convolutional neural networks in the image domain (image classification [1], target detection [2], and scene classification [3]), research on the use of convolutional neural networks for video action recognition has started to emerge, and successful applications from images to videos illustrate the superiority of convolutional neural network models. Therefore, convolutional neural network-based action recognition has become an active research area, and its algorithmic framework is shown in Figure 1.

## 2. Contribution

As an important application project of computer vision technology in the field of sports, volleyball player action standardness recognition has been a challenging research topic in the field of computer vision, and the volleyball standardness action recognition model not only requires the convolutional neural network to extract spatial information such as motion background and character appearance scene in the image but also needs to effectively extract motion information in the temporal dimension of the video. In summary, this paper combines the idea of a spatio-temporal dual channel and constructs a dual-stream DNet convolutional neural network volleyball action standardness recognition model based on Inception [4], which uses the idea of weighted fusion to connect the extracted spatio-temporal features so as to improve the training speed of the model and the accuracy of volleyball action standardness recognition. In addition to theoretical contributions, this paper also has some practical significance. As volleyball is a mainstream sport for the public, action recognition for volleyball players and volleyball fans can help improve people's ability to use volleyball and enhance the public's love for volleyball. This paper also has some general applicability and can be applied to other sports, in addition to volleyball, for action recognition.

## 3. Literature Review

**Figure 2:**
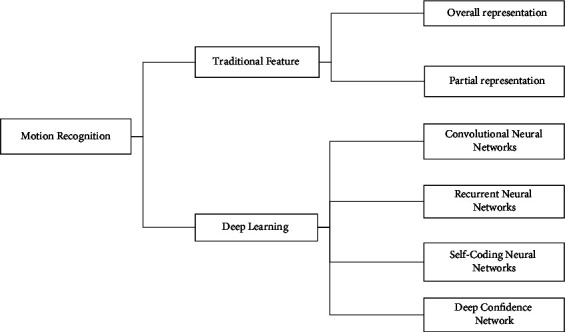
Classification of action recognition for artificial intelligence and deep learning.

For artificial intelligence and deep learning-aided volleyball movement standardization recognition models, human action recognition can be divided into behavior recognition based on traditional manual features, and behavior recognition based on depth features according to the way of feature extraction division. The specific division of behavior recognition is shown in Figure 2. Traditional feature extraction methods are generally designed manually to characterize action features through manual observation and design. Compared with traditional methods, deep learning methods do not use manual initiative to extract features, retain more valuable information in the video, and generally outperform traditional methods in terms of effectiveness. Deep learning methods applied to human behavior recognition should not only use the spatial information of the video but also the temporal information of the video, which is also the focus of the method research.

The essence of action recognition in volleyball belongs to the category of human action recognition classification. Convolutional neural networks were introduced to the video domain for action recognition. In 2014, Simonyan and Zisserman [5] proposed the first human action recognition method based on dual-stream neural networks, in which ImageNet was used to pretrain, RGB single frames and optical flow maps were used to train the dual-stream network, and finally fused for action recognition. The method makes full use of spatio-temporal feature information, improves the accuracy of action recognition, and lays a theoretical foundation for volleyball action recognition. In 2016, Wang et al. [6] proposed the time segment network (TSN), combining a sparse temporal sampling strategy with a video supervision method based on the classical dual-stream network. TSN is also composed of a spatial stream convolutional network and a temporal stream convolutional network. But unlike two-stream, which uses a single frame or a single stack of frames, TSN uses a series of short segments sparsely sampled from the entire video, each of which will give its own initial prediction of the behavior class, and the “consensus” of these segments to obtain the video-level prediction results. 2014 LSVRC challenge winner GoogleNet [7] network was proposed, with its structure of adding multiple Inception network models to the traditional deep convolutional neural network. The second deficiency is compensated by cross-practice, regularization, and data expansion techniques, and the network structure is named the temporal segmentation network (TSN). Due to the recent successful application of residual networks (ResNets) in deep network training [8], a novel spatial-temporal residual network model has been proposed that combines ResNets and a dual-flow model, which learns the spatio-temporal characteristics of the behavior hierarchically by connecting the residuals of the air-domain and time-domain flows.

## 4. Research Design

### 4.1. Convolutional Double-Stream Volleyball Action Standard Type Recognition Model Construction

For artificial intelligence and deep learning, the structure of a dual-stream DNet network can be divided into two feature extraction modes: spatial stream channel and temporal stream channel. The advantage of a DNet-based dual-stream convolutional neural network model over a traditional convolutional neural network is that it captures temporal information features in video data more clearly and efficiently. For single-frame RGB, spatial information generally expresses features mainly in the form of coordinate positions of things and fixed scenes, while for temporal information, more information about the continuous motion of the target can be conveyed in the form of the continuous motion of multiple optical flow frames. Both spatial and temporal stream channels are trained by applying the same network to the feature images, and subsequently, action recognition is achieved through spatio-temporal feature fusion.

**Figure 3:**
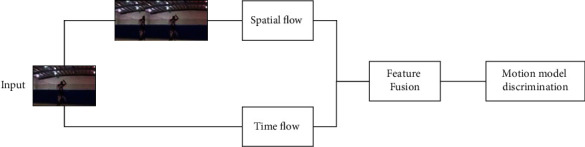
Structure of a dual-stream DNet convolutional neural network for artificial intelligence and deep learning.

The specific network structure of the dual-stream DNet network is shown in Figure 3, which uses a two-branch network architecture to capture the spatial and temporal information of the video, respectively. The air domain uses RGB images as input to extract appearance features, and the time domain uses optical flow information as input to extract temporal features, and then the two behavior recognition datasets are classified by weighted feature fusion of the two-channel features and by the multitask training method to remove overfitting and thus obtain better results.

### 4.2. GoogleNet Network Model

**Figure 4:**
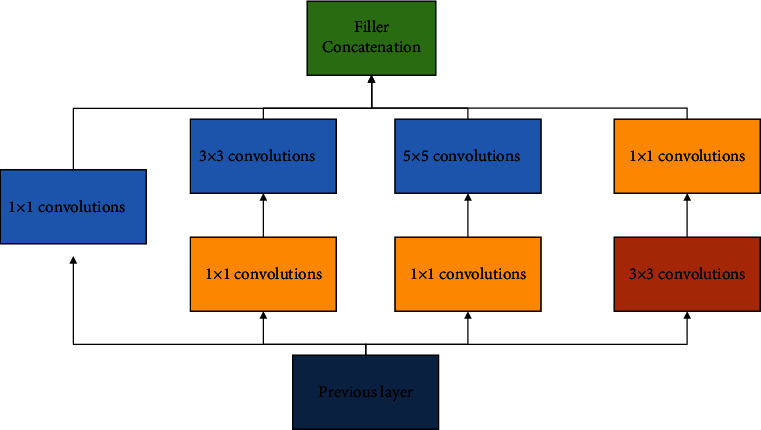
Inception module for artificial intelligence and deep learning.

With artificial intelligence and deep learning, GoogleNet is a deep neural network model based on the Inception module introduced by Google, which won the ImageNet competition in 2014. GoogleNet differs from previous networks such as AlexNet4 and VGGl6 by increasing the depth of the network to obtain better training results, which deepens the core structure of the GoogleNet network since its Inception, as shown in Figure 4. Inception is to take multiple convolutional or pooling operations and put them together to assemble a network module, and design the neural network to assemble the whole network structure in a module. In networks that do not use this approach, we often layer only one operation, such as convolution or pooling [9], and the convolution kernel size for the convolution operation is also fixed size. However, in practical situations, different sizes of convolutional kernels are needed at different scales so as to obtain the best performance. Moreover, for the same picture, different sizes of convolution kernels perform differently because they have different perceptual fields. Therefore, it is necessary to let the network choose the appropriate perceptual field by itself, and Inception can meet such a need. An Inception module provides multiple convolutional kernel operations in parallel, and the network chooses to use them by adjusting the parameters in the training process. The whole Inception structure is a series of multiple Inception modules.

The two main contributions of the Inception structure are the use of 1 × 1 convolution for lifting and dimensioning, as well as the simultaneous convolution and reaggregation at multiple dimensions. In Inception, a single convolutional kernel is changed to a model with three parallel convolutional kernels (1 × 1, 3 × 3, 5 × 5) and a single pooling layer. Each path is followed by a 1 × 1 convolution operation immediately before the convolution operation or after the pooling operation. More convolutions can be superimposed on the same size perceptual field to extract richer features, and the 1 × 1 convolution can also achieve the effect of dimensionality reduction, which reduces the computational complexity and improves the performance of the network. Therefore, in this paper, the GoogleNet network is selected as the base network for volleyball movement standardization and recognition.

### 4.3. Construction of DNet Network Model

With artificial intelligence and deep learning in this paper, we will use the improved GoogleNet, i.e., DNet, for volleyball action standardization and recognition research, and DNet optimizes the internal structure of the Inception module.

The DNet network structure improves the original three groups of parallel convolutional operations, named shallow EnInception-1 module, middle EnInception-2 module, and deep EnInception-3 module, respectively. Since the convolutional neural network with high accuracy, multiple parameters, and large computation has the problem of landing, the original convolutional structure is decoupled to improve the speed and accuracy of the deep convolutional neural network. In this paper, the original 5 × 5 convolutional structures of Inception-1 are decomposed into two 3 × 3 convolutional channels to reduce the model's computational parameters. In addition, the 3 × 3 convolutional structure of all Inceptions is modified into an asymmetric [10] convolutional pattern, i.e., two convolutional blocks of 1 × 3 and 3 × 1 are used instead of the original 3 × 3 convolutional structure to increase the stability of the model.

**Figure 5:**
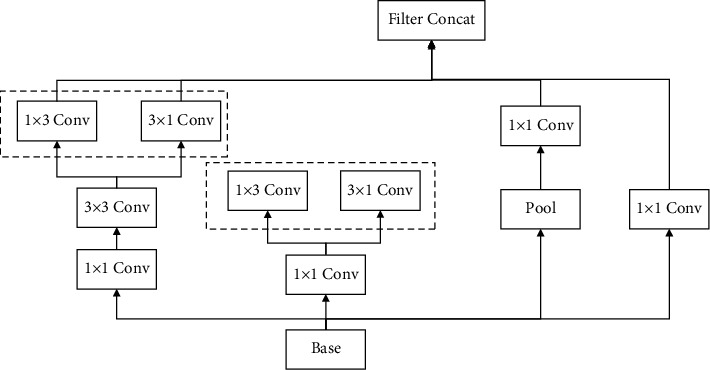
DNet Inception convolutional model for artificial intelligence and deep learning.

After improving the shallow Inception-1 module, the mid-layer Inception-2 module undergoes a larger structural improvement. Since the mid-layer module is more important for feature extraction than the shallow layer, the complete convolution operation is retained to make feature extraction more significant while accelerating the computational speed. First, the 5 × 5 sizes convolutional kernel in the traditional middle layer of the Inception-2 module structure is improved. A new convolutional layer is formed by connecting two 3 × 3 sized convolutional kernels in sequence, replacing the original large convolutional kernel structure with a new small convolutional network. The two “3 × 3” size convolutional kernels, which are closer to the output graph, are also replaced by “3 × 1” and “1 × 3” convolutional kernels in the middle Inception-2 module structure, as shown in the dashed part of Figure 5. The filter banks in the module are then extended to make the network structure wider rather than deeper, which reduces the dimensionality and results in a better performance of the neural network, which is more suitable for high-dimensional features such as the amplitude of the fine human movements in volleyball. Therefore, while the computation is faster, the accuracy of the model after adjustment is higher compared with the previous one, as shown in Figure 5. For the deeper Inception-3 structure, the original structure of the model is retained in this paper because the spatial concentration of the convolutional kernels decreases in the deeper layers of the network and the relatively larger convolutional kernels extract more abstract features and are, therefore, suitable for application in the deeper layers of the network.

In addition, an auxiliary classification layer batch is introduced to improve the convergence of the very deep network. The aim is to push useful gradients to lower layers to make them immediately useful and to improve convergence during training by resisting the vanishing gradient problem in very deep networks. The auxiliary classifier also plays the role of a regularization term. This is because the main classifier of the network performs better if the side branches are batch normalized or have a discarded layer. This also gives weak supporting evidence for the speculation that batch normalization acts as a regularization term [11].

### 4.4. Parameter Selection and Optimization

#### 4.4.1. Auxiliary Classification Layer

**Figure 6:**
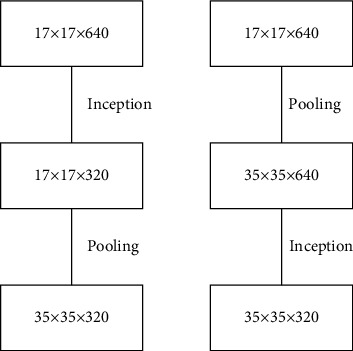
Traditional pooling method for artificial intelligence and deep learning.

**Figure 7:**
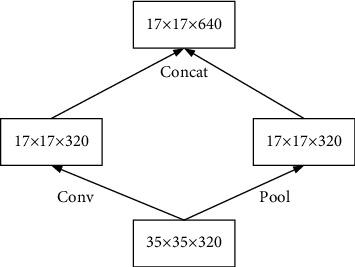
Auxiliary classification layer for artificial intelligence and deep learning.

The left panel of Figure 6 shows the traditional pooling method, which loses the information on the feature map, and the right panel shows the process of enlarging the feature map before pooling, which has the problem of computing too much brightness. In order to balance the gain and loss of both, one-half of the feature map is obtained by convolution first and one-half of the feature map is obtained by pooling, and finally stitching is performed, as shown in Figure 7. This Inception module is used for 35 × 35 down to 17 × 17 and 17 × 17 down to 8 × 8. The Inception module reduces the grid size while expanding the filter set. It is fast and avoids performance bottlenecks.

#### 4.4.2. Introduce Dropout [12] Layer and Choose the Optimal Ratio

The two improved shallow Inception modules, the five middle Inception-4 modules with optimized structure, and the two high Inception-5 modules with larger convolutional kernels were sequentially entered after the maximum pooling operation, followed by a global average pooling, and the optimal dropout ratio of 0.5 was chosen by introducing the dropout layer at the fully connected layer.

#### 4.4.3. Classification of Feature Images by AM-Softmax [13] Classifier Loses

Since this classifier has a larger class spacing and smaller intraclass distance for different action features, the AM-Softmax classifier with algorithm improvement based on Softmax [14] is chosen to be applied to volleyball action recognition, which makes the local action classification effect more obvious. The abovementioned improved DNet network for the GoogleNet network has more optimized performance compared with the original basic network.

### 4.5. Spatio-Temporal Feature Fusion Design

**Figure 8:**
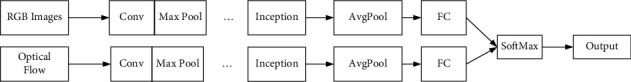
Spatio-temporal feature fusion design for artificial intelligence and deep learning.

In this paper, the spatio-temporal fusion strategy chosen for action recognition of volleyball movement in the video is designed, and the fusion approach in the classification layer is selected for feature fusion. The fusion approach for the classification layer of the dual-stream network is to perform feature fusion in the later classification layer after the fully connected layer. The dual-stream network extracts the spatial and temporal features in the motion video separately, and the weights are shared between the two networks. After convolution, pooling, and fully connected layer operations in the DNet network, the features of the two streams are fused in the classification layer, as shown in Figure 8.

In this paper, the weighted fusion method is used for feature fusion at the classification layer. For the weighted fusion method, it can be expressed as summing spatial features and temporal features after defining different weight assignments. For feature fusion, the temporal flow network and the spatial flow network are fused using the weighted fusion method with a weighted fusion weight ratio of 5 : 5 for time: space, and the fusion timing is chosen for fusion at the classification layer.

## 5. Model Regression Results and Analysis

### 5.1. Introduction to the Dataset

The experimental data in this paper are the public dataset of UCF101 [15] and the homemade volleyball sports dataset, in which the video content of the homemade dataset includes a large number of volleyball standard action demonstrations with various angles in the video, various changes in lighting information, low pixels of the video, and a short video length. The UCF101 public is an upgraded version of the UCF50 [16] database, which contains 13,320 video samples and 101 action categories. The videos in each action category are divided into 25 groups, each containing 4–7 actions. There are about 120 videos of volleyball sports in the UCF101 dataset, and there are 134 homemade datasets for VolleyballSis. All the video contents include many single volleyball basic actions and volleyball sparring actions, with various angles in the videos, various changes of lighting information, low-pixel videos, and short video length. According to the analysis requirements of the technical characteristics of volleyball standard movements, the input data are divided into four categories: (1) serving; (2) matting; (3) passing; (4) dunking.

### 5.2. Introduction to the Experimental Environment

The experimental hardware environment is shown in Table 1.

The experimental environment in this paper is the PyTorch platform, in which the input dataset UCF101 and the homemade dataset are processed in RGB and optical flow formats, and for the GNet spatial branch, RGB format images with spatial step size step = 4 are used, and for the GNet temporal flow branch, *T* = 8 continuous optical flow images are overlaid. The size of each iteration in the model training process is 100, and the learning rate is 0.001. A total of 45 iterations are performed in this experiment.

### 5.3. Evaluation Indicators

Commonly used evaluation metrics are accuracy, detection rate, and recall, where accuracy represents the proportion of samples with correct predictions, as shown in the following equation:

(1) $$\begin{array}{c} Acc=\frac{TP+TN}{N}\text{,} \end{array}$$

where TP and TN are the numbers of positive samples correctly identified as positive and negative samples correctly identified as negative, respectively, and *N* is the number of samples tested.

The detection rate is for a specific category, as shown in the following equation:

(2) $$\begin{array}{c} Precision=\frac{TP}{TP+FP}=\frac{TP}{N}\text{,} \end{array}$$

where *N* is the number of all detected target frames of a specific class, and the recall is calculated, which represents the ratio of correctly predicted target frames to all ground truths, as shown in the following equation:

(3) $$\begin{array}{c} Recall=\frac{TP}{TP+FN}\text{.} \end{array}$$

To address the different requirements for the location deviation of target detection in different scenarios, an IOU threshold is usually given, above which the detection is considered successful. As well as considering the problem of category balancing, the performance of each category is usually found separately and then averaged for each category.

First, all the detection results are sorted, with higher scores being higher, and then the detection is judged to be successful in turn. First, find the intersection ratio of detection results (dets) and real targets (gt), i.e., IOU, and find the real target with the maximum IOU value for each detection result; if this maximum IOU exceeds a certain threshold, the det is matched against this gt and considered TP, noting that each gt can only be matched once. If the IOU of a det to all gt does not exceed the threshold or the gt with the maximum IOU exceeding the threshold has been matched, it is regarded as FP. After traversing all the dets from high to low, if there is still a gt that has not been matched, it is regarded as FN.

After getting TP, FP, and FN, the precision and recall can be calculated. But different tasks have different requirements for precision and recall; some tasks require higher recall and wrong detection is acceptable, while other tasks require higher precision and missed detection is acceptable. To evaluate recall and precision as a whole, we select the first *n* of the ranked det (*n* = 1, 2, 3, ...) resulting in a recall-precision graph, and obtaining the AP value by calculating the area under this curve. The AP values for each category are averaged to obtain mAP.

### 5.4. Description of Experimental Results

The experimental results are shown in Table 2.

**Figure 9:**
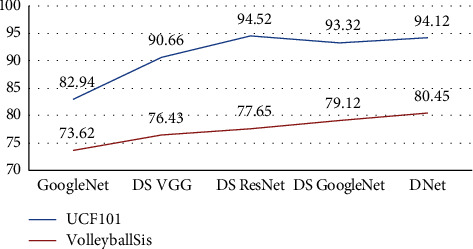
DNet accuracy comparison for artificial intelligence and deep learning.

Figure 9 shows the line graph of the DNet recognition rate, where the horizontal coordinates represent each mainstream algorithm and the vertical coordinates represent the mAP values of each algorithm on the UCF101 dataset. From the experimental results, it can be analyzed that using the weighted fusion method by setting the weight ratio to time: space 5 : 5, decoupling and heterogeneity of the GoogleNet three-layer convolutional kernel, after using the auxiliary classification layer for fusion, the average accuracy of volleyball action standardization recognition of the feature fusion dual-stream DNet convolutional neural network reached 94.12% at this time, which was higher than other network models on the 80.45% on the homemade dataset VolleyballSis, which is higher than other network models. For the model structure of the algorithm, most of the algorithm data modalities used in this paper are RGB and optical stream OF. In the to-stream structure, DNet uses RGB and OF as input, respectively. On this basis, the three-layer branching Inception structure is split, and the original 5 × 5 convolutional structure is split into two 3 × 3 convolutional kernels to reduce the computational parameters and improve the computational speed. In addition, the heterogeneous convolutional network structure is introduced, and the 3 × 3 convolutional model is further split into two convolutional models of 1 × 3 and 3 × 1 to improve the accuracy of the algorithm model without increasing the computational effort, and good results are obtained.

## 6. Conclusion and Suggestion

Artificial intelligence and deep learning have attracted much attention from researchers in industry and academia. The volleyball movement standardization and recognition model involve the application of artificial intelligence and deep learning. The research presented in this paper provides important theoretical guidance for artificial intelligence and deep learning. The heterogeneous DNet convolutional neural network with spatio-temporal feature fusion for volleyball action standardization recognition proposed in this paper greatly reduces the training parameters of the model, thus achieving the goal of improving the model speed. The introduction of heterogeneous convolution also ensures the stability problem of the model with constant parameters. It effectively improves the recognition ability and generalization ability of the model. However, there are still some problems and difficulties in volleyball action, and standardization recognition.

1. Strong dependence of the model on the motion viewpoint problem.
2. When the model is applied to the actual volleyball sports scenario, most of the captured videos are collected from random viewpoints. Therefore, in the actual application scenario of the model, the model is required to decouple the motion viewpoint and reduce the dependence problem of the model on the motion viewpoint. Nowadays, most of the data sets are focused on a single viewpoint or captured by a specific viewpoint, so the future improvement algorithm can cope with the change of complex viewpoints and handle multiple random viewpoints.
3. Weakly supervised algorithm problem.
4. Supervised learning-based behavior recognition relies heavily on the annotation of video action tags, and the annotation of video images can consume a lot of resources. With the continuous development of deep learning, the cost of video image labeling becomes more and more expensive. How to use the algorithm itself to perform image labeling has become a hot topic of research nowadays. It is based on weakly supervised or unsupervised volleyball action standard-type recognition, which can be classified without manual labeling or with only a small amount of labeling.

## Figures and Tables

**Table 1:** Hardware environment configuration for artificial intelligence and deep learning.

Environment name	Specific configuration
Memory	64 GB
OS	Ubuntu20.04.1
GPU	GeForceRTX2080Ti11G
CPU	Intel®Core™i7-10700KF
Python/PyTorch	3.8/1.8.0
Cuda	10.2

**Table 2:** Comparison of experimental results of DNet for artificial intelligence and deep learning.

Network structure	UCF101 *map* (%)	VolleyballSis *map* (%)	Input of network
GoogleNet	82.94	73.62	RGB
DS VGG	90.66	74.43	RGB + OF
DS ResNet	94.52	70.65	RGB + OF
DS GoogleNet [17]	93.32	79.12	RGB + OF
DNet	94.12	80.45	RGB + OF

## Data Availability

The data used to support the findings of this study are available from the corresponding author upon request.
